# Insanity from Chloroform

**Published:** 1850-04-01

**Authors:** 


### INSANITY FROM CHLOROFORM EMPLOYED IN PARTURITION.

During a discussion in the early part of the present session, at the Westminster Medical Society, when a paper was read by Professor Murphy, advocating the use of chloroform in midwifery, Dr. Webster, according to the reports published in the medical journals, mentioned three cases of insanity supervening after the employment of the above powerful agent during childbirth; to which he afterwards added a fourth case of the same description. Considering these examples of mental disease are interesting to the profession, and well worth recording, they are therefore transferred to the pages of The Psychological Journal.

Case I.—In this instance the patient, who had been delivered under the influence of chloroform, was, for three days subsequently, constantly incoherent and rambling. She soon afterwards became perfectly maniacal, and so furious as to require confinement in a lunatic asylum, where she remained for twelve months, when the lady was discharged cured.

Case II.—This patient never recovered from the effects of the chloroform exhibited during her confinement; and soon afterwards became quite maniacal, and continued so for many months, but she recovered ultimately.

Case III.—As this example might perhaps be considered by some psychologists not a true instance of insanity, Dr. Webster related the chief symptoms manifested by the patient, in order to remove all doubts on the subject. In the case reported, the cerebral disturbance following the exhibition of chloroform during delivery, never ceased entirely; the patient could not sleep at night for a long time, and often said she felt as if in the presence of a madman who was going to murder her. Three weeks afterwards she became almost maniacal —exhibited much mental excitement, laughing frequently; had a strong desire to sing, with other extraordinary feelings; conducted herself like an infant, and lost her memory; in which state the patient continued during five months, when recovery took place.

Case IV.—This additional illustration of the bad effects of chloroform, employed during parturition, Dr. Webster related at a subsequent meeting of the Westminster Medical Society, it having been communicated to that gentleman by a professional friend, in consequence of perusing the reports published of the three previous cases. Being a farther corroboration of the former statements respecting the powerful impressions sometimes produced by chloroform, employed during parturition, this case, like the previous, also deserves perusal. In the individual affected, only one drachm of chloroform, sprinkled upon a handkerchief, was used; but the effects it produced were so sudden and violent, that the patient, after making a deep inspiration, remained quite insensible, which greatly alarmed the attendants. With the insensibility there was likewise deadly paleness of the countenance; however, the patient slowly rallied, but had a painful and protracted labour. During several days subsequently, the lady continued in a very nervous condition, although not then actually incoherent; but she soon became so furiously maniacal, as to require coercion by a strait-waistcoat. After being insane for many months, the patient gradually recovered her reason, and ultimately got convalescent.

Considering it was only from accumulated facts and extensive experience, that sound practical knowledge respecting the employment of chloroform in midwifery could be acquired, Dr. Webster afterwards said, he related the present, as likewise the three previously narrated examples of insanity following its use, in order to contribute data towards that important object; and he availed himself of the present opportunity also to remark, that he should esteem it a favour, if other practitioners would communicate to him any well-marked case of the same kind, stating particulars which they may have met with during their practice, as he (Dr. Webster) was very desirous of collecting additional evidence upon this important subject; of course, on the express understanding, that neither the patient's name should be divulged, nor the correspondent in any way compromised; as all such communications will be considered strictly confidential in regard to individuals.

In addition to these illustrations, showing the effects of chloroform, an instance of loss of memory, after employing that remedy, has recently occurred, which appears worth mentioning in this notice; the subject being a lady who had previously enjoyed good health, and was delivered when under the influence of chloroform. For six months afterwards, the patient could not sleep at night, and her brain never seemed sound; whilst the memory continued so much impaired, that, if disturbed in the slightest degree, she could remember nothing.

Besides instantaneous death, which occasionally supervenes from the administration of chloroform, the examples just related show, that one of the consequences now ascertained to follow its use, under certain circumstances, is mental alienation; and although some practitioners may consider such attacks simply as cases of puerperal insanity, the effect is still so very serious, as to require the most attentive consideration of physicians. Stupefaction during labour, or deadly intoxication, seems the chief benefit derived from the use of chloroform in midwifery; and however desirable it may be, at first sight, considered by patients or friends to alleviate suffering, during so painful a period, nevertheless, even temporary relief from pain may be obtained at too great a sacrifice, if consequences like those now detailed should result from the employment of this powerful anæsthetic agent, which appears at present to have many proselytes, as well amongst surgeons as with some accoucheurs.

